# Prevalence of Nutrient Deficiencies Following Bariatric Surgery—Long-Term, Prospective Observation

**DOI:** 10.3390/nu17162599

**Published:** 2025-08-10

**Authors:** Maria Humięcka, Ada Sawicka, Kinga Kędzierska, Artur Binda, Paweł Jaworski, Wiesław Tarnowski, Piotr Jankowski

**Affiliations:** 1 Department of Internal Medicine and Geriatric Cardiology, Centre of Postgraduate Medical Education, 00-416 Warsaw, Polandpiotrjankowski.eu@gmail.com (P.J.); 2 Department of General, Oncological and Bariatric Surgery, Centre of Postgraduate Medical Education, 00-416 Warsaw, Poland; 3 Department of Epidemiology and Health Promotion, School of Public Health, Centre of Postgraduate Medical Education, 01-826 Warsaw, Poland

**Keywords:** bariatric surgery, nutrition assessment, anemia

## Abstract

**Background/Objectives**: To estimate the long-term prevalence of the most common nutrient deficiencies following bariatric surgery. **Methods**: Consecutive patients who underwent bariatric surgery were followed for 10 years. Anthropometric measurements, laboratory tests, and comorbidities were assessed at baseline and during follow-up visits. **Results**: A total of 155 patients were included (74.2% women; mean age 43.6 ± 9.3 years; mean body mass index [BMI]: 41.9 kg/m^2^). Patients underwent either sleeve gastrectomy (SG, *n* = 112) or gastric bypass (GB, *n* = 43). Over a median follow-up period of 10 (10–12) years, BMI decreased by 7.4 ± 5.8 kg/m^2^. In the GB group, serum iron levels decreased significantly, whereas in the SG group, a reduction was observed in total iron-binding capacity (TIBC). Serum calcium, phosphorus, and 25-hydroxyvitamin D3 levels increased during follow-up. The prevalence of deficiencies in iron (9.0% vs. 18.7%, *p* < 0.05), folic acid (1.3% vs. 11.6%, *p* < 0.001), and vitamin B12 (7.1% vs. 17.4%, *p* < 0.01) increased, while the prevalence of hypocalcemia and 25-hydroxyvitamin D3 deficiency decreased. **Conclusions**: A significant increase in the prevalence of iron, folic acid, and vitamin B12 deficiencies was observed over a 10-year follow-up after bariatric surgery. SG and GB appear to have different long-term effects on iron metabolism.

## 1. Introduction

Obesity is a growing global public health concern [1,2,3]. According to the World Health Organization, one in eight individuals worldwide was living with obesity in 2022, and the global prevalence of adult obesity has more than doubled since 1990 [2]. This unfavorable trend is particularly evident among patients with obesity-related comorbidities [4]. Contributing factors include increased availability and consumption of calorie-dense, nutrient-poor processed foods, coupled with increasingly sedentary lifestyles [2,5]. Overweight and obesity are estimated to contribute to approximately 5 million deaths annually from noncommunicable diseases, including cardiovascular disease, type 2 diabetes, cancer, neurological disorders, and chronic respiratory conditions [3].

Bariatric surgery (BS) is recognized as an effective and durable treatment for obesity, resulting in sustained weight loss and improvement or remission of obesity-related comorbidities, as demonstrated in numerous studies [6,7,8]. The number of bariatric procedures continues to increase globally [9]. Currently, sleeve gastrectomy (SG) is the most commonly performed bariatric procedure [9]. SG involves resection of the stomach to create a tubular “sleeve” along the lesser curvature [10]. Malabsorptive procedures, such as one-anastomosis gastric bypass (OAGB) and Roux-en-Y gastric bypass (RYGB), reduce nutrient absorption by bypassing segments of the gastrointestinal tract [11]. OAGB involves the creation of a gastric pouch and a single gastrojejunal anastomosis, while RYGB includes the formation of a gastric pouch, a gastrojejunal anastomosis with a 100–150 cm alimentary limb, and a jejunojejunal anastomosis with a 75 cm biliopancreatic limb [10].

However, anatomical alterations of the gastrointestinal tract following BS may lead to both macro- and micronutrient deficiencies, which can contribute to significant long-term health complications [12,13]. Therefore, lifelong monitoring and appropriate supplementation are essential components of post-bariatric care. Although short-term data on nutritional deficiencies after BS are well documented, evidence on the long-term prevalence of micronutrient deficiencies following various bariatric procedures remains limited. The aim of this study was to evaluate the prevalence of common trace element and vitamin deficiencies in patients several years after undergoing bariatric surgery, with a focus on comparing outcomes between restrictive and malabsorptive procedures.

## 2. Materials and Methods

We included consecutive patients who underwent bariatric surgery between January 2010 and January 2015. The study population consisted of individuals who had undergone either restrictive surgery (sleeve gastrectomy, SG) or malabsorptive procedures (OAGB or RYGB). The indications for surgery included age between 18 and 60 years and a body mass index (BMI) > 40 kg/m^2^ or a BMI of 35–40 kg/m^2^ in the presence of comorbidities such as metabolic or cardiovascular diseases. Patients who underwent malabsorptive surgery as a revisional procedure following a previous SG were categorized within the gastric bypass group. The follow-up assessment was conducted between September 2022 and December 2023. Every effort was made to include all eligible patients. Individuals who had died, could not be contacted, or refused to participate were excluded from the final analysis.

The study was approved by the Institutional Bioethics Committee (approval no. 87/2021), and written informed consent was obtained from all participants. At baseline and during follow-up, standardized questionnaires were used to collect data on lifestyle, comorbidities, and previous surgeries. All participants underwent anthropometric measurements, laboratory tests, 24 h ambulatory blood pressure monitoring, and body composition analysis via dual-energy X-ray absorptiometry (DXA).

Reference ranges for micronutrients were based on the American Society for Metabolic and Bariatric Surgery (ASMBS) Integrated Health Nutritional Guidelines (2016 update: Micronutrients) [14] and were defined as follows: vitamin B12 ≥ 148.0 pmol/L, folic acid ≥ 7.7 nmol/L, iron ≥ 9.0 μmol/L, ferritin ≥ 45.0 pmol/L, calcium ≥ 2.3 mmol/L, 25-hydroxyvitamin D3 ≥ 50.0 nmol/L, phosphorus ≥ 1.1 mmol/L. Micronutrient concentrations were assessed in the following numbers of participants: vitamin B12 (*n* = 155), folic acid (*n* = 154), iron (*n* = 147), ferritin (*n* = 126), calcium (*n* = 154), 25-hydroxyvitamin D3 (*n* = 137), and phosphorus (*n* = 150).

Anemia was defined according to the World Health Organization criteria: hemoglobin < 130 g/L for men and <120 g/L for women [15]. A mean corpuscular volume (MCV) between 80 and 100 fL was considered indicative of normocytosis. Hypertension was defined as self-reported use of antihypertensive medication or a 24 h systolic blood pressure ≥ 130 mmHg or diastolic blood pressure ≥ 80 mmHg. Diabetes was defined as current use of antidiabetic medication or a hemoglobin A1c level ≥ 6.5%. Hypercholesterolemia was defined as the use of statins or a low-density lipoprotein (LDL) cholesterol level ≥ 3.0 mmol/L [16]. Remission of comorbidities was defined as the presence of a condition at baseline and its absence at follow-up.

Continuous variables are presented as means with standard deviation or medians with interquartile ranges (IQR), while categorical variables are expressed as percentages. The Shapiro–Wilk test was used to assess the normality of continuous variables. Comparisons between groups were performed using the paired or unpaired Student’s *t*-test for normally distributed variables and the Mann–Whitney U test or Wilcoxon signed-rank test for non-normally distributed data. Pearson’s or Spearman’s correlation coefficients were used to assess relationships between variables, as appropriate. Bonferroni correction was applied to adjust for multiple comparisons. The multivariable logistic regression was used to identify independent predictors of micronutrient deficiencies during the follow-up. The model included the following covariates: sex, age, baseline micronutrient concentrations, type of surgery, duration of follow-up, BMI at follow-up, BMI change, and reported supplementation during the follow-up period.

The required sample sizes to achieve 80% power for detecting a difference of 4 umol/L between changes in iron concentration after 10 years of follow-up between sleeve gastrectomy and gastric bypass subgroups at a significance criterion of α = 0.05 was 40. All statistical analyses were performed using STATISTICA software, version 13.3.

## 3. Results

### 3.1. Baseline Characteristics

Out of 347 patients who underwent bariatric surgery between January 2010 and December 2014, 155 (44.7%) were re-evaluated between September 2023 and December 2024. The reasons for non-participation in the follow-up were: failure to establish contact (*n* = 132, 38.0%), refusal to participate (*n* = 34, 9.8%), severe disability or medical condition—primarily disseminated cancer (*n* = 5, 1.4%), death (*n* = 4, 1.2%; causes: COVID-19, pulmonary embolism, thyroid cancer, and unknown), and other reasons (*n* = 17, 4.9%). The patients participating and not participating in the follow-up examination did not differ significantly concerning age (43.6 ± 9.3 vs. 42.0 ± 11.6, *p* = 0.186), sex (women: 74.2% vs. 70.4%, *p* = 0.433), BMI (41.9 [IQR: 38.4–46.6] kg/m^2^ vs. 42.3 [IQR: 38.6–47.1] kg/m^2^, *p* = 0.784), and type of bariatric procedure (SG: 73.2% vs. 76.8%, *p* = 0.440).

The general characteristics of the group are presented in Table 1. The median body fat percentage was 44.7% (IQR: 40.3–47.6). Hypertension was present in 76.8% of patients, diabetes mellitus in 34.8%, and hypercholesterolemia in 63.2%.

**Figure 1:**
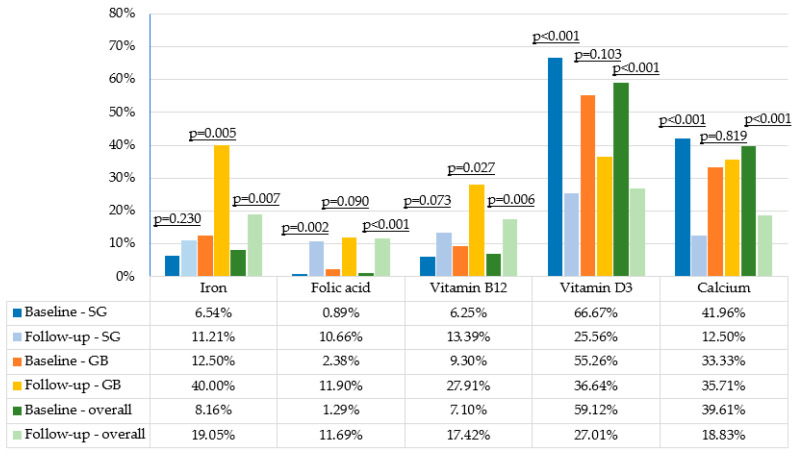
Prevalence of suboptimal nutrient concentrations and deficiencies before and after bariatric surgery, stratified by procedure type. SG—sleeve gastrectomy, GB—gastric bypass.

The most prevalent nutritional deficiency at baseline was vitamin D3 insufficiency (Figure 1). Iron deficiency occurred exclusively in women (10.8% of women vs. 0.0% of men, *p* = 0.04). Vitamin B12 and folic acid deficiencies affected only a small proportion of individuals. Anemia was diagnosed in 4 women (2.6% of the total cohort) and was not observed in any male patients.

Of the 155 study participants, 131 underwent sleeve gastrectomy and 24 underwent gastric bypass as their primary bariatric surgery. Within the SG group, 19 patients later required a revisional malabsorptive procedure, with a median interval of 3.0 years (2.0–5.0) between the primary and revisional surgeries. As a result, the final analysis was conducted on two subgroups: 112 patients who underwent SG as their sole bariatric procedure and 43 patients who underwent a malabsorptive procedure (including One Anastomosis Gastric Bypass in 33 cases and Roux-en-Y Gastric Bypass in 10 cases). Type 2 diabetes mellitus was diagnosed nearly twice as frequently among patients who underwent GB compared to those who had only SG.

### 3.2. 10-Year General Outcomes

The median follow-up period was 10.0 years (IQR: 10.0–12.0). Over this period, patients experienced a mean body weight reduction of 21.5 ± 16.2 kg and a mean BMI decrease of 7.4 ± 5.8 kg/m^2^ (Table 1). Patients in the gastric bypass group demonstrated significantly greater reductions in weight compared to the SG group (29.6 ± 18.0 kg vs. 18.4 ± 14.4 kg, *p* < 0.001), although the difference in percentage of excess weight loss (EWL) did not reach statistical significance (53.9% vs. 41.1%, *p* = 0.151). At the time of follow-up, 25.1% of all patients had achieved a BMI below 30 kg/m^2^. Overall, remission of hypercholesterolemia, type 2 diabetes mellitus, and hypertension was observed in 37.7%, 50.0%, and 29.7% of patients, respectively.

Changes in micronutrient parameters by type of surgery are presented in Table 2.

In order to identify factors associated with the occurrence of deficiencies and suboptimal concentrations of nutrients, a multivariable analysis was performed. The model included the following covariates: sex, age, baseline micronutrient concentrations, type of surgery, duration of follow-up, BMI at follow-up, BMI change, and reported supplementation during the follow-up period.

**Figure 2:**
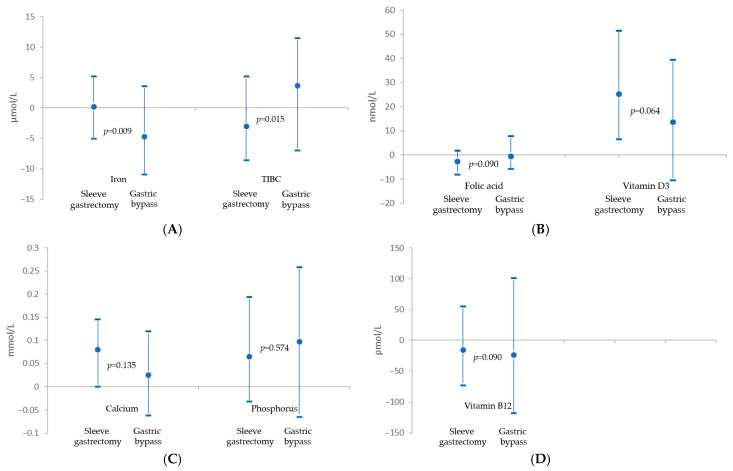
Comparison of 10-year changes in nutrient parameters between sleeve gastrectomy and gastric bypass subgroups. (**A**) Iron and TIBC (Total Iron Binding Capacity), (**B**) Folic and vitamin D3, (**C**) calcium and phosphorus, (**D**) vitamin B12. Markers represent median differences in nutritional parameter values between baseline and follow-up; whiskers indicate the upper and lower quartiles.

A significant decrease in serum iron concentration was observed exclusively in the GB group, while total iron-binding capacity (TIBC) decreased in the SG group. The changes in both iron concentration and TIBC differed significantly between the two groups (Figure 2A, Table 3). At follow-up, the median ferritin concentration was 57.1 pmol/L (IQR: 25.6–132.8) in the overall cohort, 76.2 pmol/L (IQR: 32.6–166.5) in the SG group, and 32.4 pmol/L (IQR: 18.9–53.0) in the gastric bypass group (*p* < 0.001). The median transferrin level was 37.6 μmol/L (IQR: 32.0–43.0) in the total sample, 35.4 μmol/L (IQR: 31.7–41.1) in the SG group, and 41.1 μmol/L (IQR: 37.8–47.6) in the GB group (*p* < 0.001). Median transferrin saturation was 25.5% (IQR: 16.8–33.1%) in the entire cohort, 26.3% (IQR: 19.6–33.5%) in the SG group, and 16.9% (IQR: 7.2–30.0%) in the GB group (*p* < 0.001).

Iron deficiency (ID) at follow-up was identified in 11.2% of patients in the SG group and in 40.0% of those who underwent GB (*p* < 0.001; Figure 1). Low ferritin levels were observed in 33.7% of SG patients and 61.8% of patients in the GB group (*p* = 0.005), corresponding to 41.3% of all study participants. ID was significantly more prevalent in women compared to men (23.4% vs. 7.7%, *p* = 0.039). Overall, 19.4% of female participants and 5.5% of male participants (14.8% of the total cohort) reported taking oral iron supplements; among them, 52.2% met the criteria for anemia. In multivariable analysis, undergoing GB was independently associated with a higher risk of iron deficiency (aOR: 5.44 (95% confidence interval [CI]: 2.29–12.95; *p* < 0.001). In contrast, older age was associated with a lower risk of iron deficiency (aOR per year: 0.92; 95% CI: 0.87–0.97; *p* < 0.001).

A significant change in folic acid concentration was observed only in the SG group, with a median decrease of −2.6 nmol/L (−8.2 to 1.9; *p* < 0.001; Table 2). Older age was independently associated with a lower risk of folate deficiency (aOR: 0.92; 95% CI: 0.87–0.98; *p* = 0.006). Vitamin B12 levels did not change significantly in either surgical group. The overall prevalence of vitamin B12 deficiency at follow-up was 17.4%. Both folic acid and vitamin B12 deficiencies were significantly more prevalent at follow-up compared to baseline. In multivariable regression analysis, undergoing GB was the only independent predictor of vitamin B12 deficiency (aOR: 2.50; 95% CI: 1.05–5.96; *p* = 0.037).

An overall increase in serum vitamin D concentration was observed during the follow-up period. A total of 43.4% of participants reported regular vitamin D supplementation. Supplementation rates were significantly higher among SG patients compared to those who underwent GB (47.3% vs. 23.3%, *p* = 0.007). Patients who reported vitamin D supplementation had significantly higher serum vitamin D levels at follow-up compared to those who did not: 78.1 nmol/L (IQR: 59.7–103.8) vs. 60.7 nmol/L (IQR: 36.9–86.1), *p* < 0.001. Vitamin D supplementation was the only factor independently associated with a lower risk of vitamin D deficiency (aOR: 0.18; 95% CI: 0.07–0.44; *p* < 0.001).

Serum calcium concentrations increased significantly only among patients in the SG group (Table 2), and in multivariable analysis, undergoing GB was an independent predictor of hypocalcemia (aOR: 3.89; 95% CI: 1.66–9.10; *p* = 0.002). However, the between-group difference in calcium level changes was not statistically significant (Figure 2C, Table 3). Phosphorus levels increased in both surgical groups to a similar extent (Figure 2C, Table 3).

Overall, 20.0% of participants reported regular use of a combined vitamin and mineral supplement (18.1% in the SG group vs. 25.0% in the GB group; *p* = 0.353). However, this did not significantly reduce the incidence of deficiency in any of the nutrients assessed.

Hemoglobin concentration decreased in both surgical groups over the observation period, while MCV significantly increased only in the SG group (Table 1). According to the World Health Organization criteria, anemia was identified in 30.2% of women (54.3% of whom were premenopausal) and in 15.0% of men (for prevalence in groups of women and men, *p* = 0.06). However, there was no statistically significant difference in anemia prevalence between premenopausal and postmenopausal women (35.4% vs. 22.6%; *p* = 0.139). Anemia was more prevalent in patients who underwent gastric bypass compared to those in the SG group (46.5% vs. 18.8%; *p* < 0.001).

Among anemic patients, the median mean MCV was 81.2 fL (IQR: 77.2–87.7), which was significantly lower than in patients without anemia (median MCV: 91.0 fL [IQR: 88.2–95.0], *p* < 0.001). Among those with anemia, 42.1% presented with microcytosis, while the remainder had normocytic anemia; no cases of macrocytic anemia were observed. Table 4 presents a comparison between patients with microcytic and normocytic anemia. Iron deficiency was identified in 65.9% of anemic patients, vitamin B12 deficiency in 26.8%, and folic acid deficiency in 12.2%.

In multivariate regression analysis, independent predictors of anemia at follow-up were as follows: lower baseline hemoglobin concentration (aOR: 0.72; 95% CI: 0.53–0.96; *p* = 0.026), younger age (aOR per year: 0.94; 95% CI: 0.91–0.98; *p* = 0.006), undergoing gastric bypass (aOR: 3.77; 95% CI: 1.74–8.14; *p* < 0.001), iron deficiency during follow-up (aOR: 122.63; 95% CI: 25.56–588.32; *p* < 0.000001) and iron supplementation (aOR: 4.19; 95% CI: 1.64–10.69; *p* = 0.003).

## 4. Discussion

Our study provides new data on long-term changes in trace element and vitamin levels following bariatric surgery, addressing a gap in the current literature. While numerous studies have focused on the effectiveness of bariatric procedures in terms of weight loss and comorbidity remission, long-term data on micronutrient status remain limited.

A recent meta-analysis summarizing studies with over ten years of follow-up reported an average EWL of 58.3% after sleeve gastrectomy and 56.7% after gastric bypass procedures [17]. However, some large-scale studies with extended follow-up, such as the SLEEVEPASS trial, have demonstrated more favorable outcomes for GB compared to SG (43.5% vs. 50.7% EWL) [18]. In our cohort, the weight loss outcomes were slightly lower than those reported previously. Nonetheless, our results regarding comorbidity resolution are consistent with earlier long-term observations. For example, in the Swedish Obese Subjects (SOS) study, 10 years after surgery, remission of hypercholesterolemia, type 2 diabetes, and hypertension was observed in 21%, 36%, and 19% of patient, respectively [19].

In sleeve gastrectomy, resection of the gastric fundus reduces the production of intrinsic factor and hydrochloric acid, thereby impairing the absorption of key micronutrients such as iron and vitamin B12 [11]. In addition, the associated caloric restriction may contribute to folic acid deficiency [11]. In Roux-en-Y gastric bypass, the stomach is reduced to a small pouch (approximately 20–30 mL), and food is diverted to the distal jejunum via a gastrojejunal anastomosis [20]. This results in a substantial reduction in caloric intake [20]. In one-anastomosis gastric bypass, constructing a shorter biliopancreatic limb (approximately 150 cm) has been shown to lower the risk of nutritional deficiencies while maintaining favorable bariatric outcomes [21,22].

Many patients already present with nutritional deficiencies prior to bariatric surgery, and the surgery itself may further exacerbate or lead to new deficiencies [23]. Nevertheless, preoperative screening for micronutrients has not been the routine for the majority of bariatric centers [14]. Our findings confirm that vitamin and trace element deficiencies remain a significant concern following bariatric procedures. The prevalence rates observed in our cohort are in line with those reported in the literature during medium- and long-term follow-up periods [14]. However, it is important to note that for many micronutrients and types of surgery, data on deficiency rates remain limited after such extended follow-up.

Similarly to studies with shorter follow-up periods, we observed an increased incidence of iron deficiency over the 10-year observation, particularly among patients who underwent malabsorptive procedures [14,24,25]. One of the challenges in diagnosing iron deficiency lies in the variability of its definitions. The proportion of affected patients depends on the iron status markers used and their reference thresholds. Diagnosis can be based on serum ferritin or iron levels or on a combination of tests, including transferrin saturation and TIBC. In our study, consistent with the ASMBS guidelines, iron deficiency was defined as a serum iron concentration below 50 µg/dL [14]. The preoperative prevalence of iron deficiency in our cohort was comparable to previously reported rates; for example, Caron et al. reported preoperative deficiency rates of 20.0% for iron [13,14].

Iron deficiency following bariatric surgery may result from several mechanisms, including reduced gastric acid secretion and bypassing the duodenum, which is the primary site of iron absorption [26]. Routine iron supplementation is recommended postoperatively [14], with higher doses indicated for premenopausal women due to menstrual blood loss [13,27]. Iron intake is often suboptimal after bariatric surgery compared to estimated average requirements [28]. Additionally, long-term adherence to supplementation tends to decline with time [29]. Intravenous iron therapy has been shown to be more effective than oral preparations [30].

Similarly to the Canadian study involving 537 patients, preoperative folate deficiency was rare in our cohort, likely due to folic acid fortification of food [13]. The increased incidence of folate deficiency observed postoperatively may be attributed to poor adherence to multivitamin supplementation recommendations. Dietary folate is primarily absorbed in the duodenum and proximal jejunum [31]. However, our findings did not confirm a higher incidence of folate deficiency in patients undergoing bypass procedures that exclude the upper small intestine. In contrast, consistent with other studies, we observed a reduction in folate levels following sleeve gastrectomy [32,33]. This suggests that caloric restriction and inadequate intake may play a more significant role than intestinal bypass alone.

According to the guidelines of the American Society for Metabolic and Bariatric Surgery (ASMBS), the prevalence of vitamin B12 deficiency ranges from 2% to 18% in individuals with obesity and from 4% to 20% in patients 2–5 years post–weight loss surgery [13]. Our results were consistent with this both preoperatively and in long-term follow-up. The absorption of cobalamin involves several physiological steps: after its release from food proteins by gastric acid and pepsin, vitamin B12 binds to intrinsic factor in the stomach, and this complex is subsequently absorbed in the terminal ileum [27]. ASMBS guidelines recommend lifelong vitamin B12 supplementation for all patients undergoing bariatric surgery [14]. In our long-term follow-up, the prevalence of B12 deficiency increased significantly in the whole study group, with a higher rate observed in the GB group compared to the SG group—consistent with previously published data [33]. Importantly, as confirmed by prior research, systematic supplementation can effectively prevent cobalamin deficiency [34]. Additional risk factors for vitamin B12 deficiency include a vegan diet, age over 50 years, and use of medications such as proton pump inhibitors or metformin [26].

The main manifestation of the above deficiencies is anemia [35,36,37]. In our long-term observation, the disease occurred in almost every third woman and 15% of men after bariatric surgery. Additionally, anemia occurred more than twice as often after GB than after SG. In a Portuguese retrospective cohort study involving 1999 patients who underwent bariatric surgery, 24.4% were diagnosed with anemia after four years of follow-up [38]. Women had twice the risk of developing anemia compared to men [38]. In our group the prevalence of anemia in women was not significantly higher than in men; however, younger age was associated with higher anemia risk. Therefore, it is plausible that menstruation contributed to iron deficiency, especially in the context of our predominantly female cohort. Bailey et al. analyzed data from the National Health Services database on 306 thousand patients who underwent bariatric surgery [39]. They also concluded that the risk of developing anemia was 2-fold higher after a GB than after SG [39].

Vitamin D_3_ deficiency was the most frequently observed micronutrient deficiency in our cohort, which is consistent with previous reports [12,13]. Bariatric surgery, particularly malabsorptive procedures, contributes to vitamin D deficiency by removing or bypassing segments of the small intestine responsible for its absorption. Moreover, calcium absorption is dependent on adequate vitamin D levels [40]. Interestingly, vitamin D deficiency was the only vitamin deficiency that was more prevalent before surgery than in the long-term postoperative period. This was especially evident among patients who underwent SG, in whom a postoperative increase in calcium levels was also noted. These findings may be explained by widespread and consistent vitamin D_3_ supplementation after surgery. A decline in the prevalence of vitamin D deficiency due to routine supplementation has also been reported by other authors [25]. For example, Krzizek et al., in a study conducted in Austria, observed vitamin D deficiency in 57.4% of patients three years after bariatric surgery—down from nearly universal (93.9%) deficiency preoperatively [34].

The present results underscore the necessity of implementing routine follow-up assessments for patients after bariatric procedures to enable early identification and management of micronutrient deficiencies.

This study has several limitations. These include a relatively low follow-up attendance rate, a predominance of female participants, and an overrepresentation of sleeve gastrectomy procedures. However, patient attrition over long follow-up periods is a common challenge in bariatric research, and the higher proportion of women reflects real-world trends, as women are more likely to undergo bariatric surgery [22]. SG is also currently the most commonly performed bariatric procedure worldwide [41], supporting the relevance of our cohort. Furthermore, this was a single-center analysis, which may limit generalizability.

Despite these limitations, the study has notable strengths. It features a long-term follow-up period, a well-defined and homogeneous patient population, and a prospective design. Importantly, long-term data on nutrient status following bariatric surgery remain scarce, and our findings help fill this important gap in the literature.

## 5. Conclusions

A decade after bariatric surgery, a significant increase in the prevalence of iron, folate, and vitamin B12 deficiencies was observed. Conversely, the prevalence of vitamin D deficiency decreased. The effect of bariatric surgery on iron metabolism parameters differed between patients who underwent sleeve gastrectomy and those who received gastric bypass surgery. Multivariate analysis demonstrated that gastric bypass surgery was independently associated with an increased risk of iron and vitamin B12 deficiency.

## Figures and Tables

**Table 1:** Baseline characteristics and summary of clinical outcomes.

Variable	Baseline Sleeve Gastrectomy	Follow-Up Sleeve Gastrectomy	*p*-Value	Baseline Gastric Bypass	Follow-Up Gastric Bypass	*p*-Value	Baseline Overall	Follow-Up Overall	*p*-Value
	*n* = 112	*n* = 112		*n* = 43	*n* = 43		*n* = 155	*n* = 155	
Weight, kg	119.0 (107.0–129.9)	100.2 (86.8–114.0)	<0.001	121.0 (102.5–135.0)	89.0 (75.0–100.0)	<0.001	119.0 (106.2–130.1)	95.0 (85.0–112.0)	<0.001
BMI ^1^, kg/m^2^	42.0 (38.6–46.6)	36.2 (31.4–40.3)	<0.001	41.5 (37.1–46.8)	30.8 (27.7–34.8)	<0.001	41.9 (38.4–46.6)	34.5 (29.8–39.6)	<0.001
HDL ^2^, mmol/L	1.1 (1.0–1.3)	1.6 (1.3–1.9)	<0.001	1.1 (0.9–1.3)	1.6 (1.2–1.7)	<0.001	1.1 (1.0–1.3)	1.6 (1.3–1.7)	<0.001
LDL ^3^, mmol/L	3.0 (2.4–3.5)	3.0 (2.5–3.6)	0.148	3.0 (2.3–3.4)	2.3 (1.9–2.9)	0.003	3.0 (2.3–3.5)	2.8 (2.3–3.5)	0.626
Triglycerides, mmol/L	1.6 (1.2–2.0)	1.1 (0.8–1.6)	<0.001	1.4 (1.0–2.0)	1.0 (0.8–1.4)	<0.001	1.5 (1.1–2.0)	1.0 (0.8–1.5)	<0.001
HbA1c ^4^, %	6.0 (5.4–6.4)	5.8 (5.5–6.0)	0.139	6.4 (5.6–7.9)	5.9 (5.5–6.7)	0.045	6.0 (5.4–6.7)	5.8 (5.5–6.1)	0.020
Hemoglobin, g/L	140.0 (133.0–147.0)	133.0 (124.0–145.0)	<0.001	142.0 (131.0–149.0)	125.0 (97.0–138.0)	<0.001	140.0 (133.0–148.0)	131.0 (120.0–143.0)	<0.001
MCV ^5^, fL	87.8 (85.5–90.7)	89.6 (86.7–94.2)	0.002	89.0 (85.5–92.7)	88.6 (81.0–92.5)	0.654	87.9 (85.3–90.8)	89.2 (84.9–93.6)	0.003

Continuous variables are presented as medians with interquartile ranges. ^1^ BMI—body mass index, ^2^ HDL—high-density lipoprotein, ^3^ LDL—low-density lipoprotein, ^4^ HbA1c—glycated hemoglobin, ^5^ MCV—mean corpuscular volume.

**Table 2:** Selected nutrient parameters at baseline and long-term follow-up, stratified by type of bariatric procedure.

Variable	Baseline Sleeve Gastrectomy	Follow-Up Sleeve Gastrectomy	*p*-Value	Baseline Gastric Bypass	Follow-Up Gastric Bypass	*p*-Value	Baseline Overall	Follow-Up Overall	*p*-Value
Potassium, mmol/L	4.3 (4.1–4.5)	4.3 (4.1–4.5)	0.176	4.3 (4.2–4.6)	4.2 (4.0–4.5)	0.502	4.3 (4.1–4.5)	4.2 (4.1–4.5)	0.132
Sodium, mmol/L	140.0 (139.0–142.0)	140.0 (139.0–141.0)	0.284	139.0 (138.0–141.0)	140.0 (139.0–141.0)	0.147	140.0 (139.0–141.0)	140.0 (139.0–141.0)	0.861
Iron, μmol/L	15.9 (12.9–19.7)	16.3 (12.4–21.5)	0.807	15.0 (11.1–20.8)	11.3 (5.9–18.6)	0.026	15.8 (12.5–20.1)	15.9 (10.7–20.1)	0.223
TIBC ^1^, μmol/L	62.3 (57.7–68.6)	61.8 (56.1–69.0)	0.018	67.2 (59.1–76.1)	69.5 (64.0–80.2)	0.172	64.4 (59.1–71.7)	64.2 (57.6–71.9)	0.272
Folic acid, nmol//L	16.5 (13.1–22.9)	13.4 (10.4–18.1)	<0.001	18.4 (14.5–24.9)	19.7 (12.0–30.4)	0.821	17.0 (13.6–23.1)	13.6 (10.6–21.5)	0.009
Vitamin B12, pmol/L	238.3 (197.7–323.2)	242.0 (188.9–306.2)	0.274	230.9 (180.8–290.7)	222.8 (132.8–323.9)	0.583	234.6 (194.8–301.8)	236.1 (177.1–306.2)	0.234
Calcium, mmol/L	2.27 (2.20–2.34)	2.35 (2.27–2.40)	<0.001	2.25 (2.21–2.35)	2.28 (2.22–2.37)	0.125	2.26 (2.20–2.35)	2.32 (2.25–2.40)	<0.001
Phosphorus, mmol/L	1.03 (0.97–1.16)	1.13 (1.03–1.26)	<0.001	1.10 (1.00–1.19)	1.19 (1.10–1.32)	0.003	1.06 (0.97–1.16)	1.16 (1.03–1.29)	<0.001
25-OHD ^2^, nmol/L	46.3 (36.5–24.9)	71.25 (50.8–96.0)	<0.001	47.8 (40.8–62.5)	58.0 (35.8–88.8)	<0.001	46.5 (37.5–62.5)	69.0 (46.8–67.3)	<0.001

Continuous variables are presented as medians with interquartile ranges. ^1^ TIBC—Total Iron Binding Capacity, ^2^ 25-OHD—25-hydroxycholecalciferol.

**Table 3:** Comparison of the 10-year changes in nutrient parameters between sleeve gastrectomy and gastric bypass subgroups.

Variable	Sleeve Gastrectomy	Gastric Bypass	*p*-Value
**Iron,** umol/L	0.18 (−5.01–5.19)	−4.75 (−10.9–3.58)	0.009
**TIBC ^1^,** umol/L	−3.04 (−8.6–5.19)	3.67 (−6.98–11.46)	0.015
**Calcium,** mmol/L	0.080 (0.000–0.145)	0.025 (−0.062–0.120)	0.135
**Phosphorus,** mmol/L	0.065 (−0.032–0.194)	0.097 (−0.065–0.258)	0.574
**Folic acid,** nmol/L	−2.60 (−8.16–1.93)	−0.63 (−5.67–7.93)	0.090
**Vitamin D3,** nmol/L	25.21 (6.49–51.42)	13.73 (−10.48–39.43)	0.064
**Vitamin B12,** pmol/L	−16.2 (−72.7–55.0)	−23.6 (−118.0–101.1)	0.090

Continuous variables are presented as medians with interquartile ranges. ^1^ TIBC—Total Iron Binding Capacity.

**Table 4:** Characteristics of patients with anemia divided according to erythrocyte volume.

Variable	Microcytic Anemia	Non-Microcytic Anemia	*p*-Value
**Female**	87.5%	81.8%	0.634
**Age,** years	46.9 ± 8.9	53.6 ± 8.5	0.024
**After GB ^1^**	62.5%	40.9%	0.189
**EWL ^2^**	47.5 ± 46.1%	54.5 ± 31.9%	0.581
**Hemoglobin,** g/dL	96.5 (84.0–10.95)	112.5 (109.0–117.0)	<0.001
**Iron,** mmol/L	4.5 (2.9–6.3)	8.6 (6.1–15.6)	<0.001
**Vitamin B12,** pmol/L	194.8 (108.5–249.4)	266.3 (193.3–374.1)	0.020
**Folic acid,** nmol/L	11.6 (7.7–20.6)	17.9 (12.7–24.9)	0.086

Continuous variables are presented as means with standard deviation or medians with interquartile ranges ^1^ GB—gastric bypass, ^2^ EWL—excess weight loss.

## Data Availability

The data presented in this study are available on request from the corresponding author in accordance with the data processing consent and the approval of the bioethics committee.

## Abbreviations

The following abbreviations are used in this manuscript:

aOR	Adjusted Odds Ratio
ASMBS	American Society for Metabolic and Bariatric Surgery
BMI	Body Mass Index
BS	Bariatric Surgery
CI	Confidence Interval
EWL	Excess Weight Loss
GB	Gastric Bypass
Hb_A1c_	Glycated Hemoglobin
HDL	High-Density Lipoprotein Cholesterol
ID	Iron Deficiency
IQR	Interquartile Ranges
LDL	Low-Density Lipoprotein Cholesterol
MCV	Mean Corpuscular Volume
OAGB	One-Anastomosis Gastric Bypass
RYGB	Roux-en-Y Gastric Bypass
SG	Sleeve Gastrectomy
TIBC	Total Iron Binding Capacity
WHO	World Health Organization
25-OHD	25-Hydroxyvitamin D3
